# Development and Validation of the Digital Health Literacy Questionnaire for Stroke Survivors: Exploratory Sequential Mixed Methods Study

**DOI:** 10.2196/64591

**Published:** 2025-03-25

**Authors:** Qin Ye, Wei Wang, Xuan Zeng, Yuxian Kuang, Bingbing Geng, Song Zhou, Ning Liu

**Affiliations:** 1 Department of Basic Teaching and Research in General Medicine, Department of Fundamentals Department of Nursing of Zunyi Medical University Zhuhai Campus Zhuhai China; 2 Zhuhai People's Hospital Zhuhai China; 3 Guangdong Provincial People’s Hospital Zhuhai Hospital（Zhuhai Golden Bay Center Hospital） Zhuhai China; 4 The Fifth Affiliated Hospital of Sun Yat-Sen University Zhuhai China

**Keywords:** stroke survivors, digital health literacy, validation, reliability, mixed methods study

## Abstract

**Background:**

In China, there is limited research on digital health literacy (DHL) among patients with stroke. This is mainly due to the lack of validated tools, which hinders the precision and sustainability of our country’s digital transformation.

**Objective:**

This study aimed to develop and validate a DHL scale specifically for stroke survivors in China.

**Methods:**

We used a sequential, exploratory, mixed methods approach to develop a DHL questionnaire for stroke survivors. This study comprised 418 patients with stroke aged 18 years and older. To evaluate the questionnaire’s psychometric qualities, we randomly assigned individuals to 2 groups (subsample 1: n=118, subsample 2: n=300). Construct validity was evaluated through internal consistency analysis, exploratory and confirmatory factor analyses, hypothesis testing for structural validity, measurement invariance assessments using the eHealth Literacy Scale, and Rasch analyses to determine the questionnaire’s validity and reliability.

**Results:**

This study underwent 4 stages of systematic development. The initial pool of items contained 25 items, 5 of which were eliminated after content validity testing; 19 items were subsequently retained through cognitive interviews. After an interitem correlation analysis, 2 more items were excluded, leaving 17 items for exploratory factor analysis. Finally, 2 items were excluded by Rasch analysis, resulting in a final version of the questionnaire containing 15 items. The total score range of the scale was 15-75, with higher scores indicating greater DHL competence. Results showed that principal component analysis confirmed the theoretical structure of the questionnaire (69.212% explained variance). The factor model fit was good with *χ*^2^_4_=1.669; root mean square error of approximation=0.047; Tucker-Lewis Index=0.973; and Comparative Fit Index=0.977. In addition, hypothesis-testing construct validity with the eHealth Literacy Scale revealed a strong correlation (*r*=0.853). The internal consistency (Cronbach α) coefficient was 0.937. The retest reliability coefficient was 0.941. Rasch analysis demonstrated the item separation index was 3.81 (reliability 0.94) and the individual separation index was 2.91 (reliability 0.89).

**Conclusions:**

The DHL Questionnaire for Stroke Survivors is a reliable and valid measure to assess DHL among stroke survivors in China.

## Introduction

Stroke has become one of the leading causes of death and disability in adults [1]. It is characterized by a high incidence, disability, mortality, and recurrence rate and usually results in physical disability and functional impairment [2]. Approximately 40% of patients experience functional impairment after stroke onset. In particular, more than 85% of patients with stroke experience hemiplegia, which results in impaired upper limb function and decreased motor ability [3]. Many stroke survivors have difficulty accessing outpatient treatment due to mobility issues, transport difficulties, and a lack of necessary support, which in turn affects their outcome and rehabilitation process [4].

Advances in information technology have provided new opportunities for the management of stroke survivors across time and space [5]. A study has shown that web-based health services, including health information dissemination, patient data monitoring, and remote counseling, effectively manage disease risk factors and lower stroke recurrence rates [6]. Furthermore, the internet enables stroke survivors to exercise independently after being discharged from the hospital, therefore boosting the rehabilitative impact [7]. However, the huge number and varying quality of information on the internet hamper stroke survivors’ capacity to properly access and use web-based health resources [8]. Digital health literacy (DHL) refers to an individual’s entire capacity to search for, acquire, interpret, and evaluate health information from digital sources and use it to solve health problems [9]. DHL and internet access have lately been identified as “super social determinants of health” because of their influence on broader social determinants of health [10]. DHL is required to effectively participate in the digital age and attain optimal health and well-being [11].

DHL is a process skill that develops with personal, social, and environmental settings and is impacted by personal health concerns, educational level, health state at the time of exposure to digital health resources, information-seeking motivations, and technology used [9,12,13]. DHL, like other forms of literacy, is a discursive practice that aims to uncover meaning-construction processes and essentially structures thought and behavior patterns. Its central goal is to improve the ability of individuals to fully use digital health resources for health decision-making [14].

Several eHealth literacy assessment instruments have been developed over the past 2 decades. The eHealth Literacy Scale (eHEALS) has been widely used for self-reported assessments of individuals’ abilities to use the internet [15], but it is recognized that there is a need for more comprehensive tools. The Digital Health Literacy Instrument, developed by van der Vaart and Drossaert [16], offers a broader spectrum of health 1.0 and health 2.0 skills measurement. Additionally, a comparative study by Xie and Mo [17] offered valuable information for evaluating the Digital Health Literacy Instrument and eHEALS in the older adult Chinese population. Generic tools can be used to evaluate a construct (like DHL) across a variety of populations, including healthy populations, populations with and without diseases, and populations with various diseases. The limitation is its potential insensitivity in evaluating the construct within a specific illness population, as key disease characteristics may be inadequately addressed. It is recommended that a patient population use a condition-specific instrument that focuses on content regarding clinical conditions [18].

DHL interventions, an emerging management tool for stroke survivors, have the potential to improve the quality of care provided in resource-limited settings [14]. When delivering digital interventions, clinicians must assess the patient’s level of DHL [18]. However, there is no condition-specific instrument that measures DHL specific to stroke. The purpose of this study was to develop and validate a DHL questionnaire specifically for Chinese patients with stroke, with the aim of providing scientific guidance and support for digital health interventions for patients with stroke.

## Methods

### Study Design

#### Overview

**Figure 1:**
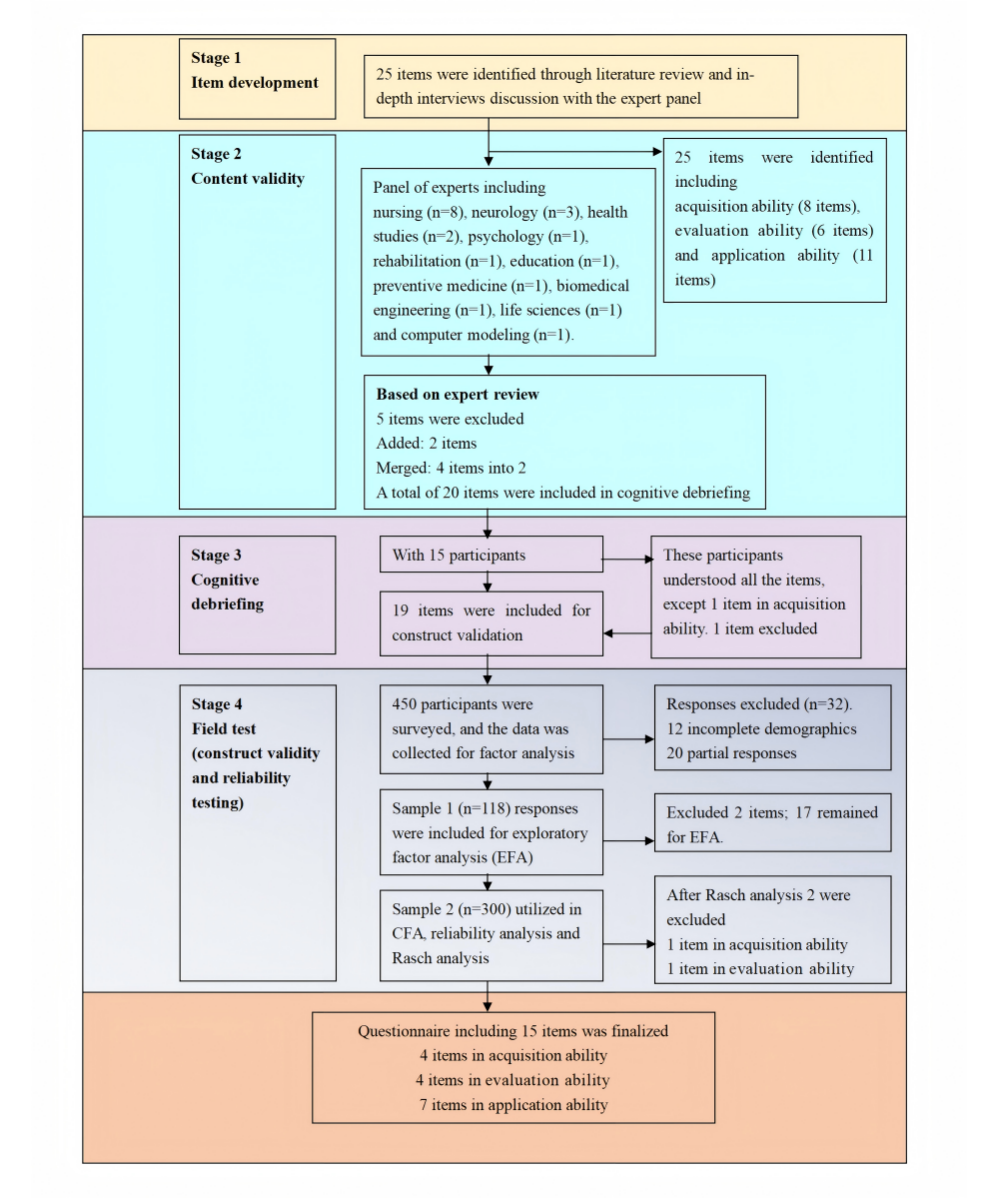
Item generation and testing process. EFA: exploratory factor analysis.

The DHL Questionnaire for Stroke Survivors was developed using a sequential, exploratory, mixed methods approach (Figure 1), and the study period ran from September 15, 2023, to April 28, 2024. The design involves an initial qualitative phase aimed at generating the item pool and a subsequent second phase aimed at testing items using quantitative techniques (psychometric evaluation).

#### Participants

We used convenience sampling, and all participants gave informed consent at the beginning. Following the survey, professional researchers conducted in-person examinations with participants. Content validation was conducted among stroke survivors recruited from the Department of Neurology and Department of Neurosurgery at the Fifth Affiliated Hospital of Zunyi Medical University, as well as among experts in the field of neurology. The inclusion criteria were defined as being aged 18 years or older, owning a smartphone, and having the ability to read and understand Chinese. Individuals with mental disorders were excluded from the study.

The field test population included survivors with self-reported strokes. Participants were aged 18 years or older and could self-complete the DHL Questionnaire for Stroke Survivors in Chinese. A minimum sample size of 250 respondents was prespecified for field testing. In Rasch analysis, a sample of 250 respondents provides 99% confidence that item calibrations and person measures are stable within a SD of 0.50 logits [19]. Psychometric procedures were conducted to determine the final items and cutoff scores of the DHL Questionnaire for Stroke Survivors.

In this research, the determination of sample size was guided by principles for conducting exploratory factor analysis (EFA). Academic literature [20] offers various heuristics for sample size estimation in EFA, with absolute minimums ranging from 100 to 250 participants [21]. Some sources suggest larger samples of 300, 500, or more [22,23]. A ratio-based approach is often recommended, where the number of participants (N) to the number of variables (p) ideally should be 5:1, meaning 5 participants for each variable [24].

For both EFA and confirmatory factor analysis (CFA), 2 separate, independent samples are necessary. The minimum required sample size for this study was calculated to be 228 participants, considering a 20% allowance for missing data. It is also noted that a sample size ranging from 30 to 200 is considered sufficient for conducting a Rasch analysis [25].

#### Conceptual Framework Definition

The lily model, grounded in social cognitive and self-efficacy theories, prioritizes enhancing capabilities and confidence over direct skill measurement to foster behavioral change and skill development [9]. We adopted the lily model to identify 3 key domains of eHealth literacy: information acquisition, evaluation, and application abilities. At its heart are 6 core skills (or literacies): traditional literacy, health literacy, information literacy, scientific literacy, media literacy, and computer literacy. Using the metaphor of a lily, the petals (literacies) feed the pistil (eHealth literacy), and yet the pistil overlaps the petals, tying them together. The lily model of eHealth literacy is used, because it is comprised of multiple literacy types, including an outline of a set of fundamental skills patients require to derive direct benefits from eHealth. A profile of each literacy type with examples of the problems patient-clients might present is provided along with a resource list to aid health practitioners in supporting literacy improvement with their patient-clients across each domain.

#### Item Generation

For item generation during the development of the new scale, it was important to pool all attributes reflecting the construct being measured. A literature review was used as the source in this study. For the comprehensive literature review, a matrix table was constructed based on the abovementioned DHL conceptualization. The first column on the left of the matrix was dedicated to the posited abilities and skills (acquire, evaluate, and apply). Beginning with the second column, the matrix incorporated specific items. From the third column onward, relevant references were systematically listed.

#### Content Validation

Content validation was conducted through a web-based expert survey designed to explore the relevance and clarity of the items and to identify items relevant to clinical concerns about DHL. The Item-Level Content Validity Index value for each item was calculated based on the proportion of expert ratings of the item’s relevance (modified κ value). Specifically, a modified κ value in the range of 0.4-0.59 indicates fair content validity, 0.60-0.74 indicates good, and ≥0.74 indicates excellent [26]. We relied on the content validity index values of the items to identify those candidates that required further in-depth exploration of their relevance and comprehensibility with stroke survivors.

#### Cognitive Debriefing

Cognitive debriefing interviews with stroke survivors were conducted face-to-face with the aim of assessing the relevance, comprehensiveness, comprehensibility, and acceptability of the DHL Questionnaire for Stroke Survivors. Verbatim transcripts of the interviews were qualitatively analyzed using thematic analysis. These stroke survivors were also invited to point out other DHL components that may have been omitted from the initial item pool.

#### Field Testing

A convenience sample of 418 participants was recruited from the Departments of Neurology and Neurosurgery of the Fifth Affiliated Hospital of Zunyi Medical University in China from December 1, 2023, to April 28, 2024.

#### Test- Retest Reliability

Of the stroke survivors who completed the study survey, 20 were asked to complete the DHL Questionnaire for Stroke Survivors again 2 weeks later.

### Measures

The eHEALS, a validated eHealth literacy measure, was used as a comparative tool to evaluate the structural validity of the DHL Questionnaire for Stroke Survivors using a hypothesis-testing method [15]. The eHEALS consists of 10 items scored on a 5-point Likert scale [15]. The eHEALS validation study showed strong internal consistency, with a Cronbach α of .88 and a test-retest dependability intraclass correlation value of 0.49 [15]. Construct validity analysis identified a single component that explained 56% of the variation [15]. The Chinese version of the eHEALS was validated using a sample of 110 high school students [27]. The Cronbach α coefficient was found to be 0.913, indicating excellent internal consistency. Factor analysis revealed loading coefficients ranging from 0.692 to 0.869. In this study, Cronbach α for the instrument was 0.93.

### Statistical Analyses

STATA (version 18; StataCorp LLC) was used to clean and prepare the data and for descriptive data analysis. We used SPSS (version 29; IBM Corp) and Amos (version 24.0; IBM Corp) for EFA and CFA. For cross-validation of structural validity, simple random sampling randomly divided the total sample into 2 subsamples. One subsample (subsample 1: n=118) was used for EFA, while the other (subsample 2: n=300) was used for CFA.

In order to determine the applicability of the subsample 1 data to the EFA, the Kaiser-Meyer-Olkin (KMO) test of sample fit and the Bartlett test of sphericity were used to determine the suitability of each category for factor analysis. In EFA, principal component analysis (PCA) was used to identify common underlying factors. Each factor was identified by eigenvalues greater than 1.0 and rotated orthogonally. A KMO value greater than 0.5 and a Bartlett *P* value less than .05 were used to assess data adequacy. CFA of subsample 2 using diagonally weighted least squares estimation. This study assessed the model fit by root mean square error of approximation<0.08, the Tucker-Lewis Index>0.95, and the Comparative Fit Index>0.95 [28].

Rasch analyses were carried out using Winsteps software (version 3.66.0; WREG). We used standard Rasch analysis procedures. First, we analyzed the fit statistics Then, we deleted items whose response patterns varied from model assumptions. Finally, we re-estimated the item parameters until they were stable.

The Rasch model, a unidimensional measurement framework, was used to assess the likelihood of endorsing items based on their difficulty and the respondents’ ability. This model assumes local independence and unidimensionality, meaning that all items are reflections of a single underlying construct [29]. Local independence was confirmed by examining item residual correlations, with correlations below 0.20 supporting the assumption [30]. Infit MnSq statistics should fall within the range of 0.5 to 1.5 for effective measurement [31]. A cutoff of Infit MnSq >1.4 with a standardized z score (*Z*_Std_) >2 can be used to identify problematic items [31]. To further evaluate the data, a person-item map was also created. An item separation index of >1.5 was considered indicative of sufficient internal consistency [32].

Measurement reliability was evaluated using person separation indices with defined thresholds: 0.67-0.80 (fair), 0.81-0.90 (good), 0.91-0.94 (very good), and >0.94 (excellent) [33]. The strata were classified as follows: 2 (poor), 2-3 (fair), 3-4 (good), 4-5 (very good), and >5 (magnificent). To confirm model fit, we ran repeated Rasch analyses, modifying items to create new scales and assessing person reliability, separation indices, and scale-to-sample targeting.

### Ethical Considerations

The Ethics Committee of Zunyi Medical University’s Fifth Affiliated Hospital (2023ZH0084) authorized the study. The study adheres to the principles of the Declaration of Helsinki, and data collection, storage, and analysis are compliant with the European Union General Data Protection Regulation. Informed consent was obtained from all participants, who received a small gift. All images in the manuscript are anonymized to protect participant identities.

## Results

### Item Development

The literature review extracted an initial pool of 25 attributes that filled the cells of the matrix table constructed in this study (Multimedia Appendix 1).

#### Content Validation

Content validity indices were calculated for each item based on expert ratings of relevance. The Item-Level Content Validity Index scores for these items ranged from 0.40 to 1, with 20 (80%) of the draft items rated as good or excellent.

The experts’ review (n=20) of the item pool informed changes to the wording of items to improve clarity (Multimedia Appendix 2).

#### Cognitive Debriefing

Cognitive debriefing was established through cognitive debriefing interviews with 15 Chinese stroke survivors aged 18 years or older. The participants’ ages ranged from 53 to 72 years, with 7 (46%) participants being male and 8 (53%) participants being older than 65 years. The average monthly income for the participants was between CNY ¥3000 and ¥5000. A total of 7 (47%) participants reported living with a spouse, while 11 (73%) participants had been living with their condition for over a year. In terms of daily activities, 13 (87%) participants demonstrated a mild level of dependence, while 2 (13%) participants were moderately dependent. It is worth noting that 3 (20%) participants were experiencing the illness for the first time.

Thematic analysis was used to classify problems with the relevance, comprehensiveness, clarity, and acceptability of the draft items. The key themes and exemplar quotations from the thematic analysis can be found in Multimedia Appendix 3. The draft DHL Questionnaire for Stroke Survivors consisted of 20 items, organized into 3 domains based on a priori theoretical classification. Each domain was constructed as an independent scale. We used a 5-point Likert scale for assessment (1=strongly disagree, 2=disagree, 3=undecided, 4=agree, and 5=strongly agree). Higher scores indicated a higher level of DHL.

### Field Testing

#### Validation Sample Characteristics

A total of 480 questionnaires were distributed, yielding 450 completed responses, resulting in a response rate of 93.75%. Of these, 32 submissions were deemed incomplete and were therefore excluded from the analyses. Consequently, the final sample comprised 418 complete responses, achieving a completion rate of 100%. The ages of the respondents varied from 33 to 90 years, with a mean age of 65.25 (SD 12.02) years. The sample consisted of 167 female respondents, accounting for 40% of the total. Table 1 provides further details.

**Table 1:** General characteristics of the study participants.

Characteristics		Subsamples, n (%)		
		1^a^ (n=118)	2^b^ (n=300)	3^c^ (n=20)
**Sex**				
	Male	66 (56)	185 (62)	13 (65)
	Female	52 (44)	115 (38)	7 (35)
**Age group (years)**				
	18-45	5 (4)	12 (4)	0 (0)
	46-65	54 (46)	11 (39)	4 (20)
	66-69	12 (10)	36 (12)	3 (15)
	70-79	33 (28)	89 (30)	8 (40)
	≥80	14 (12)	47 (15)	5 (25)
**Residence**				
	Urban	67 (57)	161 (54)	7 (35)
	Rural	51 (43)	139 (46)	13 (65)
**Education**				
	Elementary and below	47 (40)	109 (36)	6 (30)
	Middle school	27 (23)	91 (30)	5 (25)
	Secondary or vocational school	32 (27)	68 (23)	4 (20)
	University or college and above	12 (10)	32 (11)	5 (25)
**Living with children**				
	Yes	52 (44)	110 (37)	7 (35)
	No	66 (56)	190 (63)	13 (65)
**Marital status**				
	Married	90 (76)	218 (73)	15 (75)
	Unmarried, divorced, or widowed	28 (24)	82 (27)	5 (25)
**Monthly per capita household income (CNY ¥)**				
	<3000	24 (20)	46 (15)	1 (5)
	3000-5000	46 (39)	150 (50)	10 (50)
	>5000	48 (41)	104 (35)	9 (45)
**Medical cost payment method**				
	Self-paid	7 (6)	13 (4)	0 (0)
	Medical insurance	111 (94)	28 (96)	2 (100)
**First-time illness**				
	Yes	40 (34)	86 (29)	9 (45)
	No	78 (66)	21 (71)	11 (55)
**Type of stroke**				
	Hemorrhagic	22 (19)	14 (5)	0 (0)
	Ischemic	96 (81)	28 (95)	20 (100)
**Duration of illness**				
	<3 months	31 (26)	92 (31)	9 (45)
	3 months to 1 year	29 (25)	15 (50)	10 (50)
	>1 year	58 (49)	58 (19)	1 (5)
**Self-care ability**				
	Mild dependence	75 (64)	22 (76)	17 (85)
	Moderate dependence	43 (36)	70 (23)	1 (5)
	Severe dependence	0 (0)	4 (1)	2 (10)

^a^Subsample 1: exploratory factor analysis.

^b^Subsample 2: confirmatory and Rasch analysis.

^c^Subsample 3: test-retest reliability.

During the scale refining process, we first used the critical ratio method, sorted scale scores to identify high and low groups, and calculated critical ratio values using *t* tests. Item 19 was eliminated because its critical ratio value fell below the threshold of 3. Furthermore, Spearman rank correlation analysis was performed, resulting in the elimination of item 18 due to a correlation coefficient of less than 0.4. Following these analyses, the scale was simplified to include 17 items.

#### EFA Examination

Following an EFA on the 17-item subset, the KMO score was 0.901, which was above the acceptable threshold of 0.7, suggesting that the data were appropriate for component analysis. The Bartlett test of sphericity showed significant results (*χ*^2^_136_=1372.687, *P*<.001). PCA showed 3 variables with eigenvalues greater than 1, accounting for 69.212% of the variance, showing excellent explanatory power (Table 2). The scree plot confirmed the retention of 3 factors, as the line leveled off after the fourth factor (Multimedia Appendix 4). The scale’s construct validity was confirmed by factor loadings over 0.5 and no cross-loadings above 0.5. The items were divided into 3 categories: “acquisition ability,” “evaluation ability,” and “application ability.”

**Table 2:** Exploratory factor loadings of the digital health literacy questionnaire for stroke survivors (n=118).

Item		Domains		
		Factor 1	Factor 2	Factor 3
**Item 1**				
	I track stroke news and updates on the internet.	0.123	0.798	0.206
**Item 2**				
	I can search online for in-depth stroke information.	0.162	0.705	0.174
**Item 3**				
	I can find needed stroke-related info online.	0.335	0.857	0.170
**Item 4**				
	I can understand online stroke info.	0.308	0.840	0.268
**Item 5**				
	I can gather stroke data from various online platforms.	0.128	0.788	0.223
**Item 6**				
	I can check the accuracy of stroke information with medical experts.	0.180	0.324	0.761
**Item 7**				
	I verify if online stroke info is current.	0.220	0.154	0.76888
**Item 8**				
	I can validate stroke ad claims with health care staff.	0.101	0.280	0.783
**Item 9**				
	I can review expert-provided stroke information.	0.025	0.067	0.794
**Item 10**				
	I can review stroke health info online and in WeChat.	0.156	0.199	0.773
**Item 11**				
	I can discuss health issues during virtual medical sessions.	0.727	0.259	0.221
**Item 12**				
	I can participate in online discussions focused on stroke.	0.642	0.332	0.060
**Item 13**				
	I can identify stroke warning signs online.	0.771	0.148	0.141
**Item 14**				
	I can use online guides to plan my meals.	0.727	–0.027	0.037
**Item 15**				
	I can get medication info online and take meds as experts say.	0.841	0.194	0.051
**Item 16**				
	I can follow online resources for stroke exercises.	0.866	0.251	0.139
**Item 17**				
	I can record my health stats weekly with digital devices.	0.825	0.183	0.277
	Eigenvalue (characteristic root)	7.541	2.521	1.704
	Variance Contribution (%)	44.357	14.830	10.026
	Cumulative variance contribution rate %	44.357	59.186	69.212
**Item 18**				
	I don’t click on pop-up ads without thinking.	—^a^	—	—
**Item 19**				
	I won’t share my own or others’ health information on social media.	—	—	—

^a^Based on the interitem correlation analysis, items 18 and 19 were considered redundant and were deleted before exploratory factor analysis.

#### CFA Examination

CFA indicated an excellent model fit, with indices (*χ*^2^_4_=1.669; root mean square error of approximation=0.047; Tucker-Lewis Index=0.973; Comparative Fit Index=0.977) aligning with conventional criteria for a good fit. Composite reliability estimates varied from 0.861 to 0.932, and average variance extracted scores were between 0.555 and 0.664, indicating convergent validity. Furthermore, the factor loading values, which are shown in Multimedia Appendix 5, varied from 0.616 to 0.937, supporting convergent validity.

#### Reliability Testing

The value for Cronbach α, as further evidence of internal consistency reliability, was 0.937. The coefficient of test-retest reliability was 0.941, indicating a high degree of consistency in the measurements over time.

#### Hypothesis-Testing Construct Validity

The correlation coefficient between the DHL Questionnaire for Stroke Survivors and eHEALS was 0.853 (*P*<.001).

#### Rasch Analysis

To evaluate the functionality of the rating scale, we reviewed the Winsteps 3.66.0 output tables and item category probability curves for the 17 items.

After optimization of the response scales, we conducted a Rasch analysis to report the finalized characteristics of the DHL questionnaire for stroke survivors. The DHL Questionnaire for Stroke Survivors consists of 15 items. Multimedia Appendix 6 lists the items included. The Chinese version of the DHL Questionnaire for Stroke Survivors is provided in Multimedia Appendix 7. The scale meets the unidimensional criteria of the Rasch model. The first residual logarithm of the PCA of residuals ranged between 1.4 and 1.8. Item mean squared error values ranged from 0.71 to 1.38 logits, and equipment mean squared error values ranged from 0.67 to 1.75 logits. Items 4 and 7 showed ambiguity in the model. Ultimately, 15 items met the acceptable item fit criteria for the Rasch model.

Four items showed local item dependency, with residual correlation values >0.4 (range 0.44-0.59). In practical terms, a degree of local dependency is always observed in empirical data; therefore, it is necessary to consider the implications for content validity before proceeding with item removal [34]. After a qualitative appraisal of the 4 dependent pairs, we retained all items to ensure a comprehensive assessment of DHL in stroke survivors.

Last, we evaluated item separation, person reliability and separation, and internal consistency reliability. The scale had mean person ability values within a SD of 1.0 logits of mean item difficulty. Person ability ranged from −3.2 to 2.8 logits. Multimedia Appendix 8 shows the item person maps for the DHL Questionnaire for Stroke Survivors. The item separation index was 3.81 (reliability 0.94) and the individual separation index was 2.91 (reliability 0.89).

## Discussion

### Principal Findings

This study aimed to develop and validate the DHL Questionnaire for Stroke Survivors, comprising of the three components (1) acquisition ability, (2) evaluation ability, (3) and application ability, with a total of 15 items. The validation process included assessing the questionnaire’s content validity, construct validity, internal consistency reliability, test-retest reliability, and Rasch analyses. The findings demonstrate the robust psychometric properties of the DHL Questionnaire for Stroke Survivors within the target population in China. Unlike other DHL-related scales [15,16], this study specifically addresses stroke-related competencies in acquisition, evaluation, and application, emphasizing practical applications and interactions with health care providers directly pertinent to stroke management.

The eHEALS developed by Korean scholars for patients with diabetes with comorbid chronic diseases assesses “cognitive behavior” and “digital communication ability,” emphasizing the role of “cognition” as a critical component of information health literacy for patients with chronic diseases [18]. Similarly, “accessibility” in this study is closely linked to cognition, as evidenced by behaviors such as tracking stroke news and updates on the internet, searching on the web for in-depth stroke information, and gathering stroke data from various web-based platforms. These actions highlight the cognitive abilities required to identify and filter relevant information, reflecting the mental processes essential for individuals to navigate and process stroke-related information in a digital environment.

In this study, CFA and EFA were performed on 2 separate samples to evaluate the structural validity of the DHL Questionnaire for Stroke Survivors [35]. The CFA results validated the model developed through EFA. Furthermore, the CFA findings demonstrated that each item corresponded to a distinct underlying construct, and the associated item errors were uncorrelated, indicating the reliability of the CFA results [35].

In this study, a *χ*^2^_4_<3 was used as the criterion for goodness of fit [36]. The results indicated a *χ*^2^_4_=1.669, demonstrating a good model fit. Additionally, the composite reliability of all 3 factors exceeded the criterion of 0.7 [36], ranging from 0.861 to 0.932, which confirmed the good internal consistency of the items within each dimension. Furthermore, the square root of the average variance extracted for factor 1, factor 2, and factor 3 ranged from 0.555 to 0.664, surpassing the correlation values on the nondiagonal line of the discriminant validity scale, thereby further validating the model’s discriminant validity. The internal consistency of the final questionnaire was evaluated using Cronbach α, which yielded an overall value of 0.937, demonstrating the reliability and acceptability of the newly developed scale [37]. In terms of overall item performance, the individual reliability was 0.89, and the item reliability was 0.94, reflecting consistency and stability in the results. The person-item separation index was 2.91, indicating a high degree of differentiation between participants’ ability levels, while the item separation index was 3.81, signifying a high degree of difficulty differentiation among the items. The weighted mean square fit statistics (Infit MNSITEM) were 1.06 for persons and 1.07 for items, and the unweighted mean square fit statistics were 1.00 and 1.07, respectively. These values, being close to the ideal value of 1, confirm that the model fits the empirical data well.

The application of the DHL Questionnaire for Stroke Survivors represents a significant advancement in personalized health care by enabling health care providers to tailor interventions according to individual DHL levels. This approach enhances treatment adherence by equipping patients with resources and support that match their digital literacy capabilities. For example, patients with lower literacy may benefit from traditional, face-to-face interactions, whereas those with higher literacy can effectively leverage digital tools and resources [38]. Furthermore, personalized health care facilitated by the DHL Questionnaire for Stroke Survivors fosters patient engagement by ensuring that health information is both accessible and comprehensible, thereby empowering patients to take a more active role in their recovery process [39]. This not only improves the overall efficacy of rehabilitation programs but also optimizes the allocation of health care resources by targeting interventions to meet the specific needs of each patient [40].

### Limitations

This study has several limitations. First, the representativeness of the sample is limited, as the respondents were predominantly older individuals with multiple comorbidities. Second, essential items and content for assessing DHL may have been overlooked, given that DHL encompasses a wide range of skills, including complex technology-related health literacy in the digital era. Approaches to DHL have evolved significantly over the past decade and will likely continue to do so. The criterion validity of the DHL Questionnaire for Stroke Survivors was not tested due to the rapidly evolving nature of digital literacy and the absence of a gold standard for assessment. Consequently, the questionnaire should be updated to reflect advancements in this field. Third, the DHL Questionnaire for Stroke Survivors relies on self-reporting, which limits its ability to accurately capture objective proficiency. Future studies should enhance reliability by measuring the temporal stability of individual responses. Finally, the DHL Questionnaire for Stroke Survivors has only been psychometrically tested in Chinese, necessitating cross-national and cross-language validations to confirm its cultural applicability.

### Conclusion

With the rapid development of the internet and digital technology, DHL plays an increasingly important role in public health management. For patients with stroke, DHL not only affects their ability to access and understand health information but also directly influences their self-management and quality of life during rehabilitation. Therefore, it is important to develop high-quality measurement tools to assess the level of DHL among patients with stroke in order to better meet the rehabilitation needs of this population. To this end, we developed the DHL Questionnaire for Stroke Survivors. This questionnaire has good psychometric properties and is important for the development of effective health education and interventions.

## Appendix

Multimedia Appendix 1 Concept coverage matrix for digital health literacy based on a comprehensive literature review.

Multimedia Appendix 2 Item-Level Content Validity Index (I-CVI) assessment for each item in the Digital Health Literacy (DHL) Questionnaire for Stroke Survivors.

Multimedia Appendix 3 Themes and exemplar quotations from content validation of the Digital Health Literacy (DHL) Questionnaire for Stroke Survivors item pool: cognitive debriefing (n=15) and expert survey (n=20).

Multimedia Appendix 4 Scree plot analysis from exploratory factor analysis of the Digital Health Literacy (DHL) Questionnaire for Stroke Survivors.

Multimedia Appendix 5 Findings of confirmatory factor analysis for the Digital Health Literacy (DHL) Questionnaire for Stroke Survivors.

Multimedia Appendix 6 Final Digital Health Literacy (DHL) Questionnaire for Stroke Survivors—ready for use.

Multimedia Appendix 7 Chinese version of the final Digital Health Literacy (DHL) Questionnaire for Stroke Survivors.

Multimedia Appendix 8 Person-item map for the Digital Health Literacy (DHL) Questionnaire for Stroke Survivors.

## Abbreviations

CFA: confirmatory factor analysis
DHL: digital health literacy
EFA: exploratory factor analysis
eHEALS: eHealth Literacy Scale
KMO: Kaiser-Meyer-Olkin
PCA: principal component analysis

## Data Availability

The datasets generated or analyzed during this study are available from the corresponding author on reasonable request.
