# Supplementary material for: Development and Validation of the Digital Health Literacy Questionnaire for Stroke Survivors: Exploratory Sequential Mixed Methods Study
Source: J Med Internet Res. 2025 Mar 25;27:e64591. doi: 10.2196/64591 (PMC12007621; doi:10.2196/64591)
Supplement: Multimedia Appendix 1 [file jmir_v27i1e64591_app1.docx]

**Multimedia Appendix 1** Concept Coverage Matrix for Digital Health Literacy based on Comprehensive Literature Review.

| **Domain** | **Items** | Feng J（2023） | Rachmani E（2022） | van der Vaart R（2017） | Yoon J  （2022） | Norman (2006） | Paige SR（2019） | Liu HX,（2021） |
| --- | --- | --- | --- | --- | --- | --- | --- | --- |
| Acquisition ability | 1. I can express my physical and health needs. |  |  | √ |  |  | √ |  |
|  | 1. I know what stroke health information I need (e.g., early screening for stroke). | √ |  |  |  |  | √ |  |
|  | 1. I can understand stroke-related materials on the internet (e.g., drug instructions, health education materials). | √ |  | √ |  |  |  |  |
|  | 1. I can understand stroke-related medical terms on the internet, such as (thrombolytic therapy, good limb positioning, muscle strength, etc.). | √ |  |  |  |  |  |  |
|  | 1. I know where to find useful stroke-related information on the internet. |  | √ |  | √ | √ | √ | √ |
|  | 1. I obtain stroke-related health information through media such as WeChat, TikTok, Baidu, etc. | √ |  |  | √ | √ | √ |  |
|  | 1. I actively search online for the stroke rehabilitation information I need. |  |  |  | √ |  | √ |  |
|  | 1. I can filter out the stroke-related information I need from the internet. | √ |  |  | √ |  |  |  |
| Evaluation ability | 1. I can check whether the stroke-related information on the internet is up-to-date. |  |  |  | √ |  | √ |  |
|  | 1. I compare and analyze the credibility of stroke information sources on the internet. | √ | √ |  | √ |  | √ | √ |
|  | 1. I compare and analyze the credibility of stroke information sources on the internet. | √ | √ |  | √ |  | √ | √ |
|  | 1. I can judge the authenticity of stroke-related promotional advertisements on the internet. | √ |  | √ | √ | √ | √ | √ |
|  | 1. I can distinguish the quality of stroke health information on the internet. | √ |  | √ | √ | √ | √ |  |
|  | 1. I verify the authenticity of stroke information on the internet with medical staff. | √ |  |  | √ | √ | √ |  |
| Application ability | 1. I pay attention to stroke-related information on the internet. | √ |  |  | √ | √ | √ |  |
|  | 1. I know how to use the internet to answer stroke-related questions I am interested in. |  | √ |  | √ | √ | √ |  |
|  | 1. I can use stroke-related mini-programs or public accounts for self-management. | √ | √ |  | √ |  | √ |  |
|  | 1. I can provide diagnostic information needed by doctors through online consultations on Network platform (e.g., symptom description, past medical history, health indicators, etc.). | √ | √ |  | √ |  | √ |  |
|  | 1. I participate in discussions on social networks about stroke (cerebral infarction and cerebral hemorrhage) related topics. | √ | √ |  | √ |  | √ |  |
|  | 1. I use the internet to obtain stroke risk factors, such as hypertension, diabetes, hyperlipidemia, smoking, etc., and take corresponding preventive measures. | √ |  |  | √ |  | √ |  |
|  | 1. I use the internet to obtain early stroke symptom information for early self-identification and regular screening. | √ |  |  | √ |  | √ |  |
|  | 1. I use the internet to obtain dietary information and arrange my diet reasonably. | √ |  |  | √ |  | √ |  |
|  | 1. I use the internet to obtain drug-related information and adhere to correct medication. | √ |  | √ | √ |  | √ |  |
|  | 1. I use the internet to obtain rehabilitation exercise information and persist in functional exercises. | √ | √ |  | √ |  | √ |  |
|  | 1. I can use a smartphone or tablet to record and manage my physical health indicators (such as blood sugar, blood pressure, body fat, etc.). |  | √ |  | √ |  | √ | √ |

**References:**

1.Feng jiang,Jin Huang. Development of a Self-Assessment Scale for Electronic Health Literacy in Patients with Diabetes Mellitus. Chinese Journal of Nursing Education ,2023,20(1):44-48. DOI:10.3761/j.issn.1672-9234.2023.01.008.

2.Rachmani E, Haikal H, Rimawati E. Development and validation of digital health literacy competencies for citizens (DHLC), an instrument for measuring digital health literacy in the community. *Comput Methods Programs Biomed Update*. 2022;2:100082. doi:10.1016/j.cmpbup.2022.100082

3.van der Vaart R, Drossaert C. Development of the Digital Health Literacy Instrument: Measuring a Broad Spectrum of Health 1.0 and Health 2.0 Skills. *J Med Internet Res*. 2017;19(1):e27. Published 2017 Jan 24. doi:10.2196/jmir.6709

4.Yoon J, Lee M, Ahn JS, et al. Development and Validation of Digital Health Technology Literacy Assessment Questionnaire. *J Med Syst*. 2022;46(2):13. Published 2022 Jan 24. doi:10.1007/s10916-022-01800-8

5.Norman C D, Skinner H A. EHEALS: the ehealth literacy scale[J]. Journal of Medical Internet Research, 2006, 8(4): e27. DOI:10.2196/jmir.8.4.e27.

6.Paige SR, Stellefson M, Krieger JL, Miller MD, Cheong J, Anderson-Lewis C. Transactional eHealth Literacy: Developing and Testing a Multi-Dimensional Instrument. J Health Commun. 2019;24(10):737-748. doi:10.1080/10810730.2019.1666940

7.Liu HX, Chow BC, Liang W, Hassel H, Huang YW. Measuring a Broad Spectrum of eHealth Skills in the Web 3.0 Context Using an eHealth Literacy Scale: Development and Validation Study. *J Med Internet Res*. 2021;23(9):e31627. Published 2021 Sep 23. doi:10.2196/31627
