# Supplementary material for: Development and Validation of the Digital Health Literacy Questionnaire for Stroke Survivors: Exploratory Sequential Mixed Methods Study
Source: J Med Internet Res. 2025 Mar 25;27:e64591. doi: 10.2196/64591 (PMC12007621; doi:10.2196/64591)
Supplement: Multimedia Appendix 2 [file jmir_v27i1e64591_app2.docx]

**Multimedia Appendix 2** Item-level Content Validity Index (I-CVI) Assessment for Each Item in the DHL Questionnaire for Stroke Survivors.

| **Items** | **Experts** | | | | | | | | | | | | | | | | | | | |  |
| --- | --- | --- | --- | --- | --- | --- | --- | --- | --- | --- | --- | --- | --- | --- | --- | --- | --- | --- | --- | --- | --- |
|  | **1** | **2** | **3** | **4** | **5** | **6** | **7** | **8** | **9** | **10** | **11** | **12** | **13** | **14** | **15** | **16** | **17** | **18** | **19** | **20** | **I-CVI/κ** |
| **1** | 4 | 3 | 4 | 2 | 4 | 3 | 4 | 4 | 4 | 4 | 4 | 4 | 4 | 4 | 4 | 4 | 4 | 4 | 4 | 3 | 0.95 |
| **2** | 3 | 4 | 3 | 3 | 4 | 4 | 3 | 4 | 4 | 4 | 4 | 4 | 3 | 3 | 3 | 3 | 4 | 3 | 4 | 4 | 1 |
| **3** | 4 | 4 | 4 | 3 | 4 | 4 | 4 | 4 | 3 | 3 | 4 | 3 | 4 | 3 | 4 | 4 | 3 | 3 | 4 | 4 | 1 |
| **4** | 4 | 4 | 3 | 4 | 4 | 4 | 4 | 4 | 3 | 3 | 3 | 4 | 4 | 3 | 4 | 4 | 4 | 3 | 3 | 3 | 1 |
| **5** | 4 | 4 | 4 | 4 | 3 | 3 | 4 | 4 | 4 | 4 | 3 | 4 | 4 | 4 | 3 | 3 | 4 | 4 | 4 | 3 | 1 |
| **6** | 4 | 3 | 3 | 3 | 3 | 4 | 4 | 4 | 3 | 3 | 3 | 3 | 4 | 4 | 3 | 4 | 4 | 3 | 4 | 3 | 1 |
| **7** | 3 | 4 | 3 | 4 | 4 | 3 | 4 | 3 | 4 | 3 | 4 | 3 | 4 | 4 | 4 | 4 | 3 | 3 | 2 | 2 | 0.90 |
| **8** | 4 | 4 | 3 | 4 | 4 | 3 | 3 | 4 | 4 | 3 | 3 | 4 | 4 | 4 | 4 | 4 | 3 | 4 | 4 | 4 | 1 |
| **9** | 3 | 5 | 3 | 4 | 3 | 2 | 3 | 4 | 4 | 2 | 4 | 4 | 2 | 4 | 4 | 2 | 3 | 4 | 3 | 3 | 0.80 |
| **10** | 4 | 4 | 3 | 4 | 4 | 4 | 4 | 4 | 3 | 3 | 3 | 4 | 4 | 4 | 3 | 3 | 4 | 4 | 4 | 3 | 1 |
| **11** | 4 | 4 | 4 | 4 | 3 | 3 | 4 | 4 | 4 | 4 | 3 | 3 | 3 | 3 | 3 | 4 | 4 | 4 | 3 | 4 | 1 |
| **12** | 4 | 3 | 3 | 3 | 3 | 4 | 4 | 4 | 3 | 3 | 3 | 4 | 3 | 4 | 4 | 3 | 4 | 3 | 2 | 2 | 0.90 |
| **13** | 3 | 4 | 3 | 4 | 3 | 4 | 3 | 4 | 4 | 3 | 4 | 4 | 3 | 4 | 4 | 3 | 3 | 4 | 4 | 3 | 1 |
| **14** | 3 | 3 | 4 | 2 | 3 | 3 | 2 | 4 | 2 | 3 | 3 | 2 | 4 | 4 | 4 | 4 | 4 | 4 | 3 | 3 | 0.80 |
| **15** | 4 | 3 | 4 | 4 | 4 | 4 | 4 | 3 | 4 | 3 | 3 | 3 | 4 | 4 | 3 | 3 | 3 | 4 | 4 | 4 | 1 |
| **16** | 4 | 4 | 4 | 3 | 3 | 3 | 4 | 4 | 4 | 3 | 4 | 4 | 3 | 4 | 3 | 4 | 4 | 3 | 4 | 4 | 1 |
| **17** | 3 | 4 | 4 | 4 | 4 | 3 | 3 | 3 | 3 | 3 | 4 | 4 | 3 | 3 | 3 | 4 | 4 | 3 | 3 | 3 | 1 |
| **18** | 4 | 4 | 4 | 3 | 3 | 3 | 4 | 3 | 4 | 4 | 4 | 4 | 4 | 4 | 3 | 4 | 4 | 3 | 4 | 4 | 1 |
| **19** | 3 | 4 | 3 | 4 | 3 | 4 | 4 | 3 | 4 | 4 | 3 | 4 | 4 | 4 | 4 | 4 | 4 | 3 | 3 | 3 | 1 |
| **20** | 4 | 4 | 4 | 3 | 3 | 3 | 3 | 4 | 4 | 4 | 3 | 3 | 3 | 3 | 4 | 3 | 4 | 4 | 3 | 3 | 1 |
| **21** | 2 | 1 | 2 | 3 | 4 | 2 | 3 | 2 | 2 | 4 | 2 | 4 | 2 | 2 | 3 | 1 | 4 | 2 | 3 | 1 | 0.4 |
| **22** | 3 | 2 | 4 | 4 | 3 | 2 | 2 | 4 | 2 | 3 | 1 | 1 | 1 | 4 | 4 | 2 | 1 | 2 | 2 | 2 | 0.4 |
| **23** | 4 | 2 | 4 | 2 | 2 | 3 | 2 | 3 | 1 | 1 | 1 | 3 | 1 | 1 | 3 | 1 | 3 | 3 | 4 | 4 | 0.5 |
| **24** | 2 | 3 | 2 | 3 | 1 | 3 | 1 | 4 | 2 | 4 | 4 | 1 | 4 | 4 | 2 | 1 | 2 | 2 | 2 | 2 | 0.4 |
| **25** | 4 | 3 | 1 | 4 | 2 | 2 | 3 | 2 | 2 | 4 | 4 | 2 | 4 | 2 | 4 | 2 | 3 | 2 | 3 | 2 | 0.5 |
