# Supplementary material for: Development and Validation of the Digital Health Literacy Questionnaire for Stroke Survivors: Exploratory Sequential Mixed Methods Study
Source: J Med Internet Res. 2025 Mar 25;27:e64591. doi: 10.2196/64591 (PMC12007621; doi:10.2196/64591)
Supplement: Multimedia Appendix 3 [file jmir_v27i1e64591_app3.docx]

**Multimedia Appendix 3** Themes and exemplar quotations from content validation of the DHL Questionnaire for Stroke Survivors item pool: cognitive debriefing (n = 15) and expert survey (n = 20).

| **Theme** | **Exemplar quote** |
| --- | --- |
| Content relevance | “...With so much information out there on the internet, you have to search and discern for yourself. This scale checks if we can do that properly, which I think is pretty good.”– Participant 10 |
| Content comprehensiveness | “It covers a lot of ground, from looking up information to asking doctors, participating in online discussions, and researching medication. However, I think it could also include some questions about using health apps on our phones, like those for measuring blood pressure or sugar levels, which are quite important for us at this age.”– Participant 8 |
| Item clarity | “Most questions are clear enough, but some are a bit tricky, like verifying if the information found online is up-to-date. How are we, old folks, supposed to do that? Maybe someone should explain what some of these terms mean.”– Participant 1 |
| Item acceptability | “The scale is okay, not too long, and doesn't have too many questions. I find it easy enough to fill out. Some questions make you think, but that's also a good thing; it makes you reflect on your habits.”– Participant 6 |
| Response scales | “The 1 to 5 scoring, I think it's just right. But sometimes I feel like I'm neither very compliant nor very non-compliant; I'm somewhere in between. I wish the options were a bit more nuanced.”– Participant 3 |
