# Supplementary material for: Development and Validation of the Digital Health Literacy Questionnaire for Stroke Survivors: Exploratory Sequential Mixed Methods Study
Source: J Med Internet Res. 2025 Mar 25;27:e64591. doi: 10.2196/64591 (PMC12007621; doi:10.2196/64591)
Supplement: Multimedia Appendix 4 [file jmir_v27i1e64591_app4.docx]

**Multimedia Appendix 4** Scree Plot Analysis from Exploratory Factor Analysis of the DHL Questionnaire for Stroke Survivors.


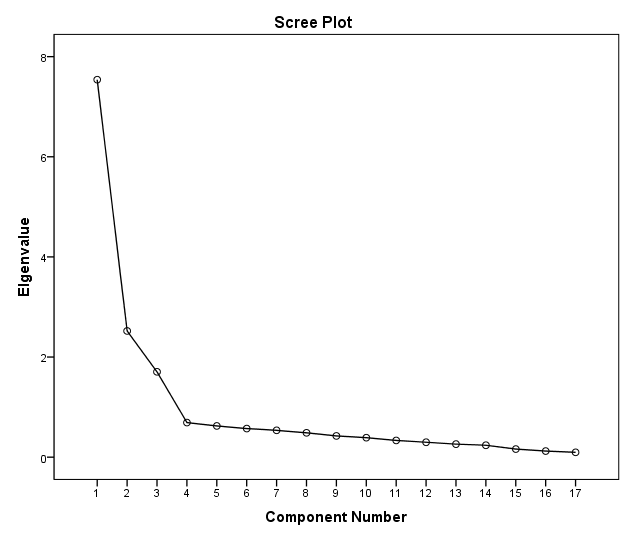


^a^EFA: Exploratory factor analysis
