# Supplementary material for: Development and Validation of the Digital Health Literacy Questionnaire for Stroke Survivors: Exploratory Sequential Mixed Methods Study
Source: J Med Internet Res. 2025 Mar 25;27:e64591. doi: 10.2196/64591 (PMC12007621; doi:10.2196/64591)

**Multimedia Appendix 5** Findings of confirmatory factor analysis for the DHL Questionnaire for Stroke Survivors.


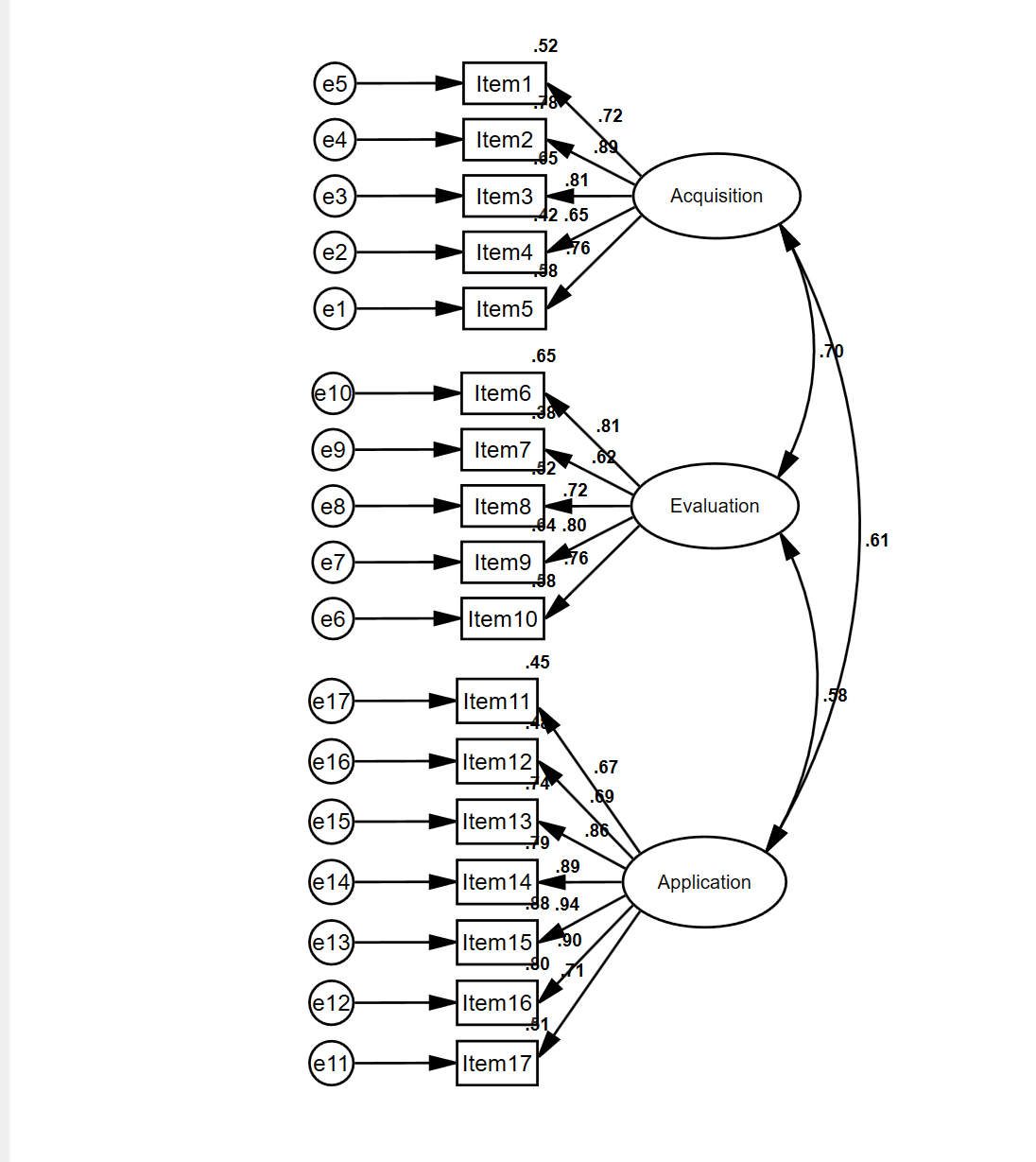

Supplement: Multimedia Appendix 5 [file jmir_v27i1e64591_app5.docx]
