# Supplementary material for: Development and Validation of the Digital Health Literacy Questionnaire for Stroke Survivors: Exploratory Sequential Mixed Methods Study
Source: J Med Internet Res. 2025 Mar 25;27:e64591. doi: 10.2196/64591 (PMC12007621; doi:10.2196/64591)
Supplement: Multimedia Appendix 6 [file jmir_v27i1e64591_app6.docx]

**Multimedia Appendix 6** Final DHL Questionnaire for Stroke Survivors – Ready for Use.

| **Domain** |  | **Items** | **Degree of Congruence** | | | | |
| --- | --- | --- | --- | --- | --- | --- | --- |
|  |  |  | Strongly Agree | Agree | Undecided | Disagree | Strongly Disagree |
| Acquisition ability | Item 1 | I track stroke news and updates on the internet. | 5 | 4 | 3 | 2 | 1 |
|  | Item 2 | I can search online for in-depth stroke information. | 5 | 4 | 3 | 2 | 1 |
|  | Item 3 | I can find needed stroke-related info online. | 5 | 4 | 3 | 2 | 1 |
|  | Item 4 | I can gather stroke data from various online platforms. | 5 | 4 | 3 | 2 | 1 |
| Evaluation  ability | Item 5 | I can check the accuracy of stroke information with medical experts. | 5 | 4 | 3 | 2 | 1 |
|  | Item 6 | I can validate stroke ad claims with healthcare staff. | 5 | 4 | 3 | 2 | 1 |
|  | Item 7 | I can review expert-provided stroke information. | 5 | 4 | 3 | 2 | 1 |
|  | Item 8 | I can review stroke health info online and in WeChat. | 5 | 4 | 3 | 2 | 1 |
| Application ability | Item 9 | I can discuss health issues during virtual medical sessions. | 5 | 4 | 3 | 2 | 1 |
|  | Item 10 | I can participate in online discussions focused on stroke. | 5 | 4 | 3 | 2 | 1 |
|  | Item 11 | I can identify stroke warning signs online. | 5 | 4 | 3 | 2 | 1 |
|  | Item 12 | I can use online guides to plan my meals. | 5 | 4 | 3 | 2 | 1 |
|  | Item 13 | I can get medication info online and take meds as experts say. | 5 | 4 | 3 | 2 | 1 |
|  | Item 14 | I can follow online resources for stroke exercises. | 5 | 4 | 3 | 2 | 1 |
|  | Item 15 | I can record my health stats weekly with digital devices. | 5 | 4 | 3 | 2 | 1 |
