# Supplementary material for: Development and Validation of the Digital Health Literacy Questionnaire for Stroke Survivors: Exploratory Sequential Mixed Methods Study
Source: J Med Internet Res. 2025 Mar 25;27:e64591. doi: 10.2196/64591 (PMC12007621; doi:10.2196/64591)
Supplement: Multimedia Appendix 7 [file jmir_v27i1e64591_app7.docx]

**Multimedia Appendix 7** Chinese Version of the Final DHL Questionnaire for Stroke Survivors.

| **测试条目** | **相符程度** | | | | |
| --- | --- | --- | --- | --- | --- |
|  | 非常  符合 | 比较符合 | 不确定 | 比较  不符合 | 非常  不符合 |
| 1.1我会关注网上中风相关的信息 | 5 | 4 | 3 | 2 | 1 |
| 1.2我会主动上网搜索中风相关信息 | 5 | 4 | 3 | 2 | 1 |
| 1.3我能够查到我需要的中风相关信息 | 5 | 4 | 3 | 2 | 1 |
| 1.4我会积极寻找多种途径获取中风相关信息 | 5 | 4 | 3 | 2 | 1 |
| 2.1我会向医护人员咨询网上中风信息来源的可靠性 | 5 | 4 | 3 | 2 | 1 |
| 2.2我能通过向医护人员判断网上与中风相关宣传广告的真伪 | 5 | 4 | 3 | 2 | 1 |
| 2.3我能够判断不同途径提供的中风信息的专业程度 | 5 | 4 | 3 | 2 | 1 |
| 2.4我会谨慎地评估网络推送或微信群里的中风健康信息 | 5 | 4 | 3 | 2 | 1 |
| 3.1我会使用在线问诊，向医生描述病情 | 5 | 4 | 3 | 2 | 1 |
| 3.2我会参与网上（如微信群里）关于中风的讨论 | 5 | 4 | 3 | 2 | 1 |
| 3.3我能利用网络资源对中风早期症状进行识别 | 5 | 4 | 3 | 2 | 1 |
| 3.4我能利用络获取的饮食相关信息，合理安排饮食 | 5 | 4 | 3 | 2 | 1 |
| 3.5我能上网查询药物信息，在听取专家意见后正确服药 | 5 | 4 | 3 | 2 | 1 |
| 3.6我能利用网络获取的中风康复资料，坚持功能锻炼 | 5 | 4 | 3 | 2 | 1 |
| 3.7我会每周使用数字设备（如手机、平板等）记录我的血糖、血压等 | 5 | 4 | 3 | 2 | 1 |
