# Supplementary material for: Development and Validation of the Digital Health Literacy Questionnaire for Stroke Survivors: Exploratory Sequential Mixed Methods Study
Source: J Med Internet Res. 2025 Mar 25;27:e64591. doi: 10.2196/64591 (PMC12007621; doi:10.2196/64591)
Supplement: Multimedia Appendix 8 [file jmir_v27i1e64591_app8.docx]

**Multimedia Appendix 8** Person-item map for the DHL questionnaire for stroke survivors.


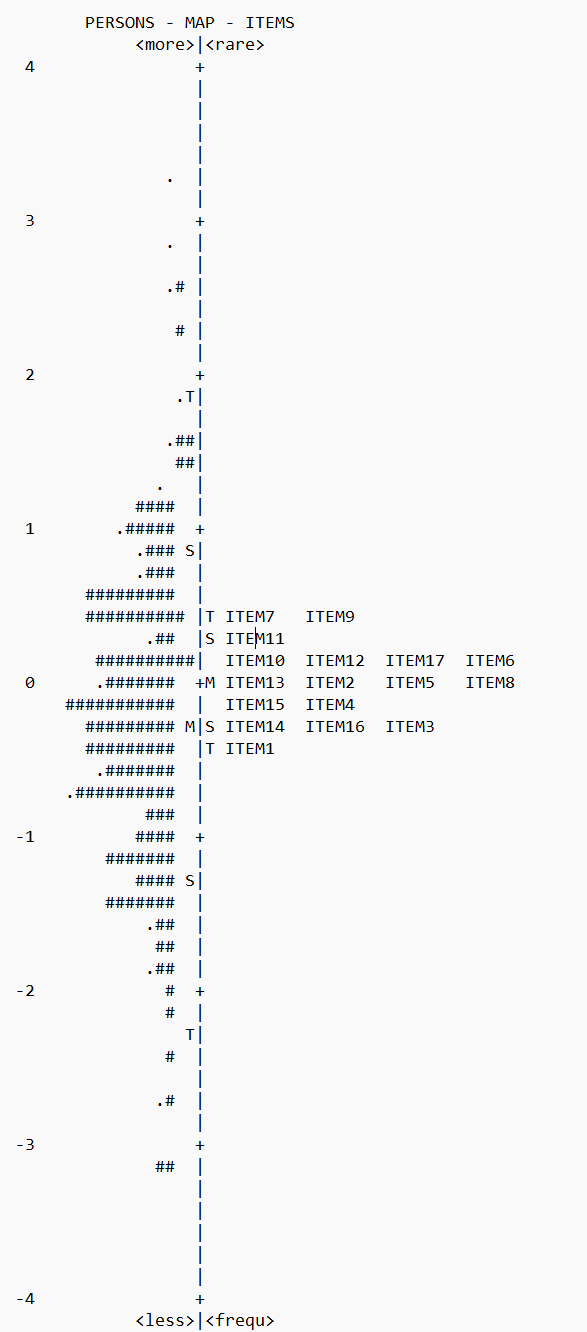


^a^ Patients are represented on the left of the dashed line by the symbol "#" (which represents 2 subjects) and "." (which indicates 1 subject). On the right of dashed line are illustrated the items of the DHL questionnaire for stroke survivors 15-item version with their number (Pnumber of item). M indicates the mean measure (on the lef the person ability and on the right the item difculty). S shows one standard deviation from the mean and T denotes two standard deviations. Higher ability for persons (higher digital health literacy) and more diffcult items are on the top of the figure.
